# Facilitating adoption of AI in natural disaster management through collaboration

**DOI:** 10.1038/s41467-022-29285-6

**Published:** 2022-03-24

**Authors:** Monique M. Kuglitsch, Ivanka Pelivan, Serena Ceola, Mythili Menon, Elena Xoplaki

**Affiliations:** 1 Fraunhofer Institute for Telecommunications, Heinrich Hertz Institute, Einsteinufer 37, 10587 Berlin, Germany; 2 Alma Mater Studiorum Università di Bologna, Department of Civil, Chemical, Environmental, and Materials Engineering, Viale del Risorgimento 2, 40136 Bologna, Italy; 3 International Telecommunication Union, Place des Nations 1211, 1202 Genève, Switzerland; 4 Department of Geography and Center for International Development and Environmental Research, Justus Liebig University Giessen, Senckenbergstrasse 1, 35390 Giessen, Germany

**Keywords:** Natural hazards, Geography, Climate-change mitigation

## Abstract

Artificial intelligence can enhance our ability to manage natural disasters. However, understanding and addressing its limitations is required to realize its benefits. Here, we argue that interdisciplinary, multistakeholder, and international collaboration is needed for developing standards that facilitate its implementation.

Acute events of natural origin (e.g., atmospheric, hydrologic, geophysical, oceanographic, or biologic) can result in disruption and devastation to society, nature, and beyond^1,2^. Such events, which disproportionately impact certain regions (e.g., least developed countries^3^) and populations (e.g., women and children^4^), are often referred to as natural disasters by experts in the geoscience and disaster risk reduction communities, as reflected in the scientific literature and in Sustainable Development Goals 11.5 and 13.1.

Recently, interest has grown in leveraging innovative technologies such as artificial intelligence (AI) to bolster natural disaster management^5^. In many fields, such as medicine and finance, AI has gained traction due to advances in algorithms, a growth in computational power, and the availability of large data sets. Within natural disaster management, it is hoped that such technologies can also be a boon: capitalizing on a wealth of geospatial data to strengthen our understanding of natural disasters, the timeliness of detections, the accuracy and lead times of forecasts, and the effectiveness of emergency communications.

This Comment looks at successes and limitations of data collection methods and AI development for natural disaster management. It then examines the challenges and solutions surrounding AI implementation. It is shown that, although AI has the promise to enhance our ability to manage natural disasters, its effective adoption depends on collaborative efforts to address these challenges.

## Successes and limitations to data

The foundation of any AI-based approach is high-quality data. A recent success is the emergence of new (and novel use of traditional) data collection methods. For example, sensor networks now help us to gather data from topographically complex regions, which are otherwise difficult to monitor, at high spatiotemporal resolutions. Such networks have proven successful for flash flood^6^ and avalanche^7^ monitoring. Although satellite-derived imagery has long been used for Earth observations, it is now being used in innovative ways. Global luminescence (i.e., nightlights) is being used by scientists to derive quantitative information about flood exposure^8^ and, with AI, can improve probabilistic scenarios of flood exposure. Through combining Global Navigation Satellite System data with AI, scientists have been able to predict tsunami amplitudes without characterizing the triggering earthquake^9^; avoiding issues such as magnitude saturation, which is common in seismic-based detection systems.

However, a number of limitations and/or technical issues must be considered when curating data for AI-based algorithms. Some of these relate to data quantity, such as: Are the data sufficient and representative? How are they stored and shared? Other concerns relate to data quality, such as: Do the data require calibration or correction? Do they have the desired spatiotemporal resolution? Are independent data available for testing the algorithm? When using AI to detect extreme events such as avalanches or earthquakes, the availability of data can be a limiting factor. AI-based methods can be very effective if a training dataset covers very large events. However, the availability of such data is limited because of the rarity of these events. One solution is producing synthetic data, which are based on a physical understanding of these hazards. Alternatively, it is possible to use machine learning algorithms requiring as few as one training event^10^. Another approach is applying transfer learning; a model is trained using data from a certain site and fine-tuned for another site^11^. Sometimes sufficient data are available, but there could be an issue with the spatiotemporal resolution. For instance, flood researchers have detected biases in numerical weather predictions (NWP) of precipitation in Japan, which can be ascribed to the smooth topography that is intrinsic in such algorithms. Rather than producing a higher-resolution NWP (which is computationally costly), these experts have turned to AI to correct these biases and produce a more accurate flood prediction^12^.

## Successes and limitations to AI development

If high-quality datasets are available, AI-based algorithms can be used to detect or forecast events by combining multiple data sources or modeling techniques. For instance, seismic source and propagation modeling can be combined in a deep learning algorithm to generate probabilistic forecasts of earthquake shaking levels at a given location^13^. In another example, automatic weather station and snowpack data can be coupled in a random forest algorithm to forecast avalanche danger with human-level accuracy^14^.

However, also at the modeling phase, there are limitations to consider. For instance, is this the best model architecture given the intended use of the algorithm? How should we evaluate the algorithm and what level of explainability do we require? What are our expectations for generalizability (e.g., is our algorithm transferable to other regions where the availability of data might be limited)? In the earthquake example, the AI-based algorithm was evaluated using two earthquake sequences (in Italy and Japan) at different shaking thresholds. It was shown that this algorithm outperformed classical earthquake detection models for most of the shaking thresholds^13^. In the aforementioned avalanche example, the AI-based algorithm agreed with human forecasts in 80% of the cases. Although a false alarm rate would have been desirable, it was not possible to compute as the avalanche danger level is based on a complex combination of many factors—including snowpack and weather—and cannot be directly measured.

Answering such questions is nontrivial because of the diverse ways that AI-based methods are employed to predict natural disasters. These differences can, for example, be ascribed to the hazard type, algorithm type, and overall objective of the algorithm. There do, however, seem to be certain basic requirements that should be met when training and testing an AI-based algorithm. However, no clear guidelines or standards exist to support researchers/developers and those evaluating or implementing the end products (e.g., policy-makers/governments, individuals/consumers, and humanitarian organizations).

## Challenges and solutions to AI implementation

Once an AI-based algorithm has been shown to accurately detect (e.g., in the avalanche example) or forecast (e.g., in the flood example) natural disasters, how can we ensure that it will be implemented to support natural disaster management? First, we need to address the disconnect between people developing the AI-based algorithms and people intended to implement them.

Often, these AI-based algorithms are developed by geoscience or machine learning experts in an academic setting (university or research institute) in order to advance the scientific understanding of a natural hazard. Throughout the lifetime of a research project, from funding acquisition to dissemination of outcomes, interaction with stakeholders and end users (including governmental emergency management agencies and humanitarian organizations) is often limited. For instance, once a project is completed, the results are shared at scientific conferences, in specialized committees, and in peer-reviewed publications, rarely reaching the aforementioned stakeholders and end users. This disconnect hinders the adoption of these AI-based algorithms.

Unfortunately, operating in a silo is not limited to geoscience and machine learning experts in an academic setting. Non-academic organizations dealing with DRR will also need an open-mindedness to new technologies and interaction with other experts (including the geoscience and machine learning experts in an academic setting) and stakeholders to reap the benefits of improved detection and forecasting for informed decision-making.

An example of an effective cross-sectoral collaboration is the Operation Risk Insights platform from IBM. This AI-based platform, which has been implemented since 2019, was developed by machine learning experts at IBM in close collaboration with end users from humanitarian organizations. These partnerships, which occurred at all stages of product development, streamlined the adoption of the platform.

Several programs are already championing interdisciplinary, multi-stakeholder, and international approaches. In the Resilient America Program, future projects will explore how new sources of data, for example, social media, can be combined with AI for predictive analysis. The European Union’s CLINT project brings together experts and stakeholders from nine countries and various sectors (national hydrometeorological services, agencies, universities, non-governmental organizations, and industry) to explore how AI can enhance climate services to support policy-makers and the interplay between research and impact. The African Union’s Africa Science and Technology Advisory Group (Af-STAG) on DRR actively liaises with experts on the continent and abroad to explore, for instance, how new data sources like street-level imagery can be combined with AI to improve the transmission of risk information to end users. Af-STAG-DRR has also engaged with the International Telecommunication Union (ITU), World Meteorological Organization (WMO), and UN Environment Programme (UNEP) Focus Group on AI for Natural Disaster Management (FG-AI4NDM), which is laying the groundwork for standards in the use of AI to support natural disaster management. This Focus Group is unique within the standardization landscape because of the diversity of its participants (including geoscientists, AI/ML specialists, DRR experts, governments, industry, and humanitarian organizations from around the globe), which ensures that a multitude of perspectives is considered.

## Interdisciplinary collaboration for the future

As we have shown, novel data sources and AI-based methods show great promise in improving the detection, forecasting, and communication of natural disasters. However, their implementation is often hindered by limited interaction between developers and implementers of AI-based solutions, and a lack of clear guidelines for those developing, evaluating (or regulating), and implementing these technologies.

To address the former, we advocate:

- expanding the participation in scientific conferences and specialized committees to include experts from relevant disciplines and non-academic stakeholders (including humanitarian organizations and governments),
- predicating research funding on partnerships with end users, and
- supporting national and international efforts to strengthen these partnerships.

For the latter, we believe that expert-produced, stakeholder-vetted, and internationally recognized standards can provide assurances that innovative technologies are applied in an informed manner with careful consideration of the limitations, and can be invaluable for supporting capacity building.
